# Evaluation of Sensor Configurations for Robotic Surgical Instruments

**DOI:** 10.3390/s151027341

**Published:** 2015-10-27

**Authors:** Jesús M. Gómez-de-Gabriel, William Harwin

**Affiliations:** 1 Departamento de Ingeniería de Sistemas y Automática, Universidad de Málaga, Calle Dr. Ortiz Ramos, 29071 Málaga, Spain; 2 School of Systems Engineering, University of Reading, Whiteknights, RG6-6AY Reading, Berkshire, UK; E-Mail: w.s.harwin@reading.ac.uk

**Keywords:** virtual sensors, force feedback, teleoperation

## Abstract

Designing surgical instruments for robotic-assisted minimally-invasive surgery (RAMIS) is challenging due to constraints on the number and type of sensors imposed by considerations such as space or the need for sterilization. A new method for evaluating the usability of virtual teleoperated surgical instruments based on virtual sensors is presented. This method uses virtual prototyping of the surgical instrument with a dual physical interaction, which allows testing of different sensor configurations in a real environment. Moreover, the proposed approach has been applied to the evaluation of prototypes of a two-finger grasper for lump detection by remote pinching. In this example, the usability of a set of five different sensor configurations, with a different number of force sensors, is evaluated in terms of quantitative and qualitative measures in clinical experiments with 23 volunteers. As a result, the smallest number of force sensors needed in the surgical instrument that ensures the usability of the device can be determined. The details of the experimental setup are also included.

## 1. Introduction

Robotics and computers can enhance minimally-invasive surgery (MIS) techniques by scaling movements, filtering micro-tremors, providing access through difficult orifices and allowing greater pre-surgical planning. Today’s robotic-aided minimally-invasive surgery (RAMIS) uses specialized and small devices often teleoperated or operated collaboratively by the surgeon. Forces encountered during contact can be measured and provide the surgeon with the sense of touch [1] and information about the health of the tissues. Likewise, although the operating time may be longer, force feedback can reduce unintentional tissue injuries [2,3], and it has been shown that tactile feedback reduces grasping force in robot-assisted surgery [4].

Related work on force-feedback biomedical applications has shown the importance of haptic feedback [5], in needle and implant insertion control [6], dentistry education [7], diagnostics through teleoperated palpation [8] and identification of abnormalities in soft tissues using rolling force sensors [9]. However, the effective use of haptic feedback in current surgical robots is still considered a challenge [10].

For reasons of size, cost, biocompatibility and sterilizability, the forces applied to the patient would ideally be estimated without using force sensors [5]. For low inertia and friction robots, forces may be estimated with limited fidelity, as there are dynamic forces that mask the relatively small force of interaction with the patient [11]. Besides, the design of surgical instruments with force feedback is under strong constraints of the size, the kind and the number of sensors and actuators, which have to be kept at a minimum for the intended task. It remains difficult to implement tactile sensors and displays compatible with the surgical environment. However force-only feedback is easier to obtain, to process and display, with advantages over visual-only feedback [12].

Different force sensors and probes have been developed for laparoscopic surgery and intraoperative diagnostics [13]. In [14], a micro-sensor composed of a central silicon post and a piezoresistor-embedded polyimide diaphragm was developed. New trends in medical robotics suggest new methods that enable sensor-less haptic teleoperation [15], which are still under development.

Analytical models of the instrument mechatronics are used for *a-priori* optimization of the teleoperated instruments. In [16], design optimization in a surgical robot system for MIS has been presented using *a-priori* knowledge of the insertion points and meta-heuristics. However, making a prototype requires advanced manufacturing capabilities, special materials and components and time.

Although certain aspects of the human operator behaviour can be modelled, such as the traditional speed-accuracy trade-offs characterized using Fitts’s law [17], these simple models of hand movements do not characterize more complex interactions, such as touch, let alone the full human behaviour. The design optimization is a complex task, which must consider human behaviour, should be accomplished by evaluating the whole task performance. The user-centred design (UCD) methodology ([18]) is based on the iterative evaluation of a product by a typical user performing a typical task. This can be expensive, unless a virtual prototyping system is used to evaluate its quality.

To enable interaction with a virtual prototype, a haptic device and a virtual reality simulator are used. The user interaction forces can be computed based on the geometry (meshes) of the manipulated objects [19] or using models with physics information to simulate the object’s behaviour while the user performs a task [20].

The virtual prototyping of surgical instruments is more complex because the environment is very dynamic (tissue elasticity, organ displacements, resections, *etc.*) and often includes special interaction forces during operations, such as bone cutting [21] or suturing.

**Figure 1:**
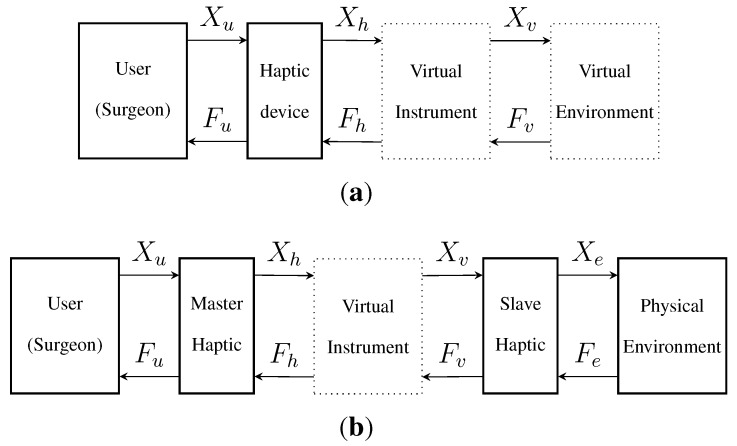
Conventional virtual prototyping method (**a**) compared to the proposed approach (**b**), where the virtual instrument is used in a real environment, reducing the complexity and inaccuracy of the simulation. (**a**) Virtual prototyping includes simulation models of the instrument and the environment; (**b**) with the proposed method, the simulated virtual instrument is used in a physical environment.

In this paper, we describe an approach based on virtual prototyping, for the evaluation of different sensor configurations of a surgical instrument. The virtual instrument has a dual interaction with the physical world, as shown in Figure 1: the user operates the simulated instrument using a master haptic device, and the simulated instrument motions are projected on a physical environment using a second (slave) haptic interface. The complexity of modelling the environment dynamics is avoided. This virtual prototyping system has been used in trials to choose the appropriate force-sensor number needed for a force-reflecting laparoscopic grasper with intraoperative diagnostic capabilities.

The remainder of the document is organized as follows. The next section describes the virtual prototyping method. Section 3 covers the experimental setup, the design and implementation of the force sensors and the set of proposed configurations. The clinical experiments are presented in Section 4. The quantitative and qualitative results are given and discussed in Section 5. Finally, conclusions about the approach and its value in this context are given.

## 2. The Virtual Prototyping Approach

A special feature of this virtual prototyping approach is its ability to reproduce the behaviour of a simulated instrument in a physical environment. For this purpose, a set of instrumented fingers mimics the actions of the ends of a simulated instrument and provides the simulator with physical force information.

**Figure 2:**
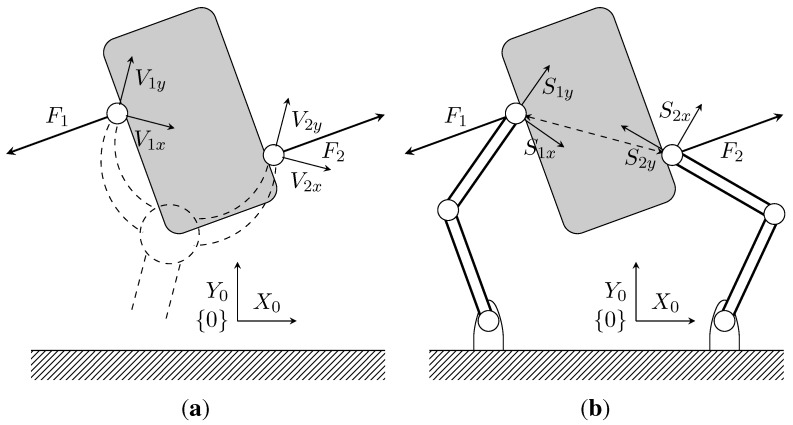
Two-dimensional representation of a physical gripper with force sensors along directions $S_{ix}$ and $S_{iy}$ and a virtual gripper with virtual force sensors along the directions of $V_{ix}$ and $V_{iy}$. (**a**) Virtual gripper; (**b**) physical gripper.

An example of its application is the simulation of a two-fingered grasper, where a human uses a haptic device to operate a virtual instrument and manipulates a physical object (see Figure 2). Even though a two-fingered virtual grasper (n=2) is being considered, this method can be extended to other types of instruments and different numbers of fingers. The virtual gripper location is defined by the centres of the physical fingertips and depends on its actual geometry. Figure 2b shows the corresponding virtual gripper concept. The positions of the finger tips coincide with the positions of the physical fingers; however, the orientation of the virtual sensors may be different, and the measured forces have to be transformed to obtain the virtual gripper forces.

### 2.1. The Virtual Instrument

**Figure 3:**
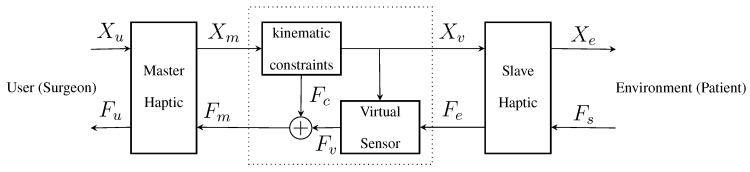
General scheme of the virtual prototyping system, where a virtual instrument, between the master and the slave haptics, is implemented by the motion constraints and virtual sensor blocks.

The virtual prototype of the instrument has two main parts, as shown in Figure 3: a kinematic constraints block and a virtual sensor block. The kinematic constraint block provides force feedback to the master haptic to keep the virtual instrument in valid position ranges. The virtual sensor block computes the readings of the virtual sensor, based on the instrument position and the physical force readings.

The input Cartesian positions $X_{m}$ from the master haptic are the desired positions of the instrument ends. The position of each end $X_{mi}$ is coerced by its kinematic constraints and then used as a reference $X_{v}$ for the slave haptics. The kinematic constraint forces can be modelled as a high-stiffness spring between the position of the master haptic interaction points (HIPs) and the closest valid point for the instrument end position.

The Cartesian forces $F_{e}$ from the physical force sensors are projected over the virtual force sensors, which also depend on the virtual instrument position $X_{v}$. Virtual sensor readings are then rotated to compensate for the virtual sensor orientation and to obtain the Cartesian virtual forces $F_{v}$. Finally, kinematic constraint forces are added to the sensed forces to provide the virtual instrument force feedback to the master $F_{m}$.

### 2.2. Virtual Sensors

The virtual sensor block of a virtual instrument is defined as a sensitivity matrix *S* that relates the sensed physical forces $F_{s}$ and the position of the virtual instrument $X_{v}$ to the corresponding virtual forces $F_{v}$ according to:

(1) $$F_{v}=R_{v(X_{v})}S{R_{v(X_{v})}}^{T}R_{s(X_{v})}F_{s}$$

Force vectors $F_{s}$ and $F_{v}$ are the composite force vectors for the *n* sensors. For the n=2 case:

(2) $$F_{s}=\left(\begin{array}{c} F_{s1}\\ F_{s2} \end{array}\right)=\left(\begin{array}{c} F_{s1x}\\ F_{s1y}\\ F_{s1z}\\ F_{s2x}\\ F_{s2y}\\ F_{s2z} \end{array}\right)\;F_{v}=\left(\begin{array}{c} F_{v1}\\ F_{v2} \end{array}\right)=\left(\begin{array}{c} F_{v1x}\\ F_{v1y}\\ F_{v1z}\\ F_{v2x}\\ F_{v2y}\\ F_{v2z} \end{array}\right)$$

The sensitivity matrix *S* in Equation (3) is a composition of the individual sensitivity matrix for each combination of virtual and physical sensors $S_{ij}$. In the case of a two-fingered instrument with a single load cell that measures the closing force as the average of the measured forces along the *x* axis direction of the two fingers:

(3) $$S=\left(\begin{array}{cc} S_{11} & S_{12}\\ S_{21} & S_{22} \end{array}\right)=\left(\begin{array}{cccccc} \frac{1}{2} & 0 & 0 & -\frac{1}{2} & 0 & 0\\ 0 & 0 & 0 & 0 & 0 & 0\\ 0 & 0 & 0 & 0 & 0 & 0\\ -\frac{1}{2} & 0 & 0 & \frac{1}{2} & 0 & 0\\ 0 & 0 & 0 & 0 & 0 & 0\\ 0 & 0 & 0 & 0 & 0 & 0 \end{array}\right)$$

The rank of the sensitivity matrix defines the number of independent force gauges needed to implement the prototype, which is an important optimization parameter.

The rotation matrix $R_{s(X_{v})}$ is a composition of the 3×3 rotation matrices for the orientation of the physical sensors, which depends on each finger position $X_{vi}$ and its kinematics.

(4) $$R_{s(X_{v})}=\left(\begin{array}{cc} R_{s1(X_{v1})} & 0\\ 0 & R_{s2(X_{v2})} \end{array}\right)$$

The rotation matrices $R_{v(X_{v})}$ and ${R_{v(X_{v})}}^{T}$ define the direct and inverse rotation of the virtual sensors, which depend on the virtual instrument position $X_{v}$ and are defined as:

(5) $$R_{v(X_{v})}=\left(\begin{array}{cc} R_{v1(X_{v1})} & 0\\ 0 & R_{v2(X_{v2})} \end{array}\right)\;{R_{v(X_{v})}}^{T}=\left(\begin{array}{cc} {R_{v1(X_{v1})}}^{T} & 0\\ 0 & {R_{v2(X_{v2})}}^{T} \end{array}\right)$$

### 2.3. Master and Slave Haptics

It is appropriate to consider haptic display devices in two classes, back-drivable impedance and admittance [22]. Back-drivable impedance-type haptics (common haptics) are designed to have low friction losses and low inertia links, so that the actuator forces are a good estimate of joint torques and, hence, endpoint forces. Admittance-type haptics are small robots with local position control and external force sensors, so regular actuators can be used. In the proposed application, an impedance-type haptic is used for the master haptic, and an admittance-type haptic is used for the slave, providing low inertia to the user and a reliable measurement of the environmental forces.

## 3. Experimental Setup

A two-fingered haptic master workstation and a slave robot with two force-sensing ball-tipped fingers have been used to implement the proposed virtual prototyping approach.

### 3.1. The Master Workstation

**Figure 4:**
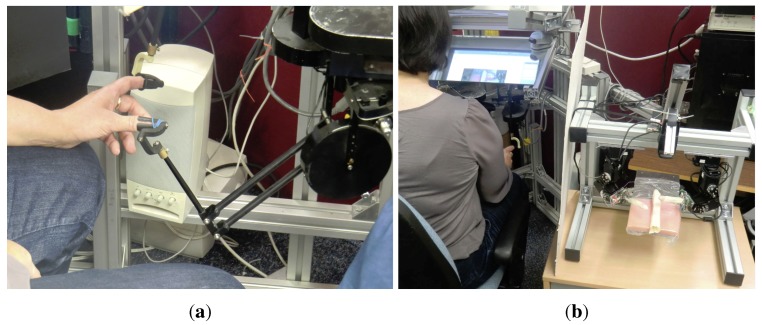
The virtual prototyping system for the evaluation of the sensor configurations of a surgical instrument. (**a**) Detail of the master multi-finger haptic workstation; (**b**) the master and the slave haptics.

Although custom solutions for multi-finger haptics [23] have been developed, two commercial haptics (Phantom Premium 1.0) were used because they have high responsiveness to human inputs. Each haptic device has a passive spherical joint at the tip (sometimes termed a finger gimbal (see Figure 4a). Its programming library includes the necessary kinematic transformations, so inputs and outputs are in the Cartesian space.

Given that tactile receptors in the human fingers can detect signals up to 10 kHz [22], to render rigid contacts (high frequency), a sampling frequency of 20 kHz might be considered necessary. However pragmatic limitations, such as the motor-amplifier response time and the characteristics of the master linkage, mean that this bandwidth is not implemented, even in specialized designs. Instead, a conventional 1-kHz update frequency was used, which is adequate for this evaluation considering contacts with soft materials.

### 3.2. The Slave System

The slave system (admittance-type haptic) consists of two small robots with local position control and a three-axis force sensor at the tip, where a 14-mm ball acts as the contact point with the environment. For the convenience of manufacturing, the slave was constructed to be between 2- and 10-times larger than a practical RAMIS. Considering that *in vitro* experiments can be performed in an open space, the shape and size of the slave is not relevant.

The final implementation of the robot was built using Dynamixel EX-106+ robot servos (Robotis Ltd., Seoul, South Korea), which features optical encoders for position feedback with 12 bits of angular resolution (about 0.07${}^{\circ }$) and low backlash. It has metal gears, aluminium links and produces a torque of about 10 Nm. Each servo has its own local position and speed control.

Communication with the finger servos uses a proprietary communication protocol over a half-duplex RS-485 serial bus with daisy-chain connections. At the maximum communication speed of 2 Mbps, using broadcast messages (multicast with no answer), the update rate for the joint position references for all six servos is over 1 kHz. With a USB to UART adapter, the average round-trip time was 1.1 ms, with a maximum response time of 5 ms on a Windows-XP operating system.

**Figure 5:**
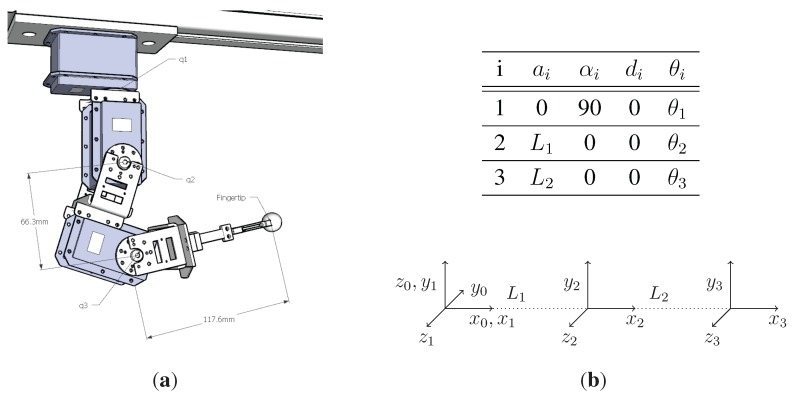
Slave haptic design and the parameter table used for the kinematic modelling according to the D–H convention. (**a**) CAD model of the slave fingers; (**b**) D–H parameter table and frames.

According to the Denavit–Hartenberg (D–H) convention, the set of reference frames and the parameter table shown in Figure 5b have been identified. The direct kinematic model is provided by the composite homogeneous transformation matrix of the last frame ${}^{0}T_{3}\left(\theta \right)$:

(6) $${}^{0}T_{3}\left(\theta \right)=\left(\begin{array}{cccc} c_{23}c_{1} & -s_{23}c_{1} & s_{1} & c_{1}\left(L_{2}c_{23}+L_{1}c_{2}\right)\\ c_{23}s_{1} & -s_{23}s_{1} & -c_{1} & s_{1}\left(L_{2}c_{23}+L_{1}c_{2}\right)\\ s_{23} & c_{23} & 0 & L_{2}s_{23}+L_{1}s_{2}\\ 0 & 0 & 0 & 1 \end{array}\right)$$

where $s_{i}$ is a compact notation for $\sin(\theta_{i})$, and similarly, $c_{23}$ is $\cos(\theta_{2}+\theta_{3})$.

### 3.3. Physical Sensors

**Figure 6:**
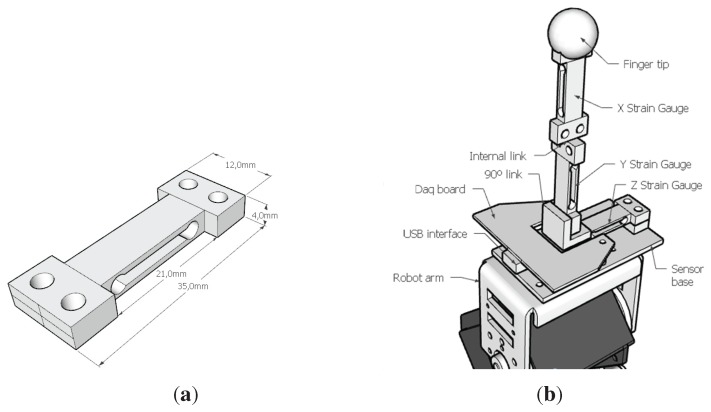
The physical force sensor has been built using three load cells and a specialized data acquisition board. (**a**) Micro-load cell dimensions; (**b**) three-axis force sensor design.

Each slave finger has a three-axis force sensor built from three standard micro-load cells in an orthogonal arrangement, as shown in Figure 6. The micro-load cells (Model CZL639HD from Bonad Tech. Ltd., Shenzhen, China and rated for 750 g) consisted of a machined aluminium frame with opposed strain gauges for temperature compensation and connected in a Wheatstone bridge configuration.

The data acquisition electronics (DaQ), previously developed for haptic devices at the University of Reading [7], were placed close to the load cells to reduce cabling and noise. It provides three-channel 12-bit ADC values through a dedicated high-speed serial port over USB. Two low-latency self-timed DaQs with a stream of 1 kS/s each were used simultaneously.

Once the load cells had been integrated into the three-axis sensor, experiments were conducted to compute the zero offsets and sensitivity matrices. In the following, example data from one of the sensors is used to illustrate the process. To determine the gravity effects, measurements were recorded in the no-contact condition, for different sensor elevation angles (see Table 1). The sensor Y-axis is always horizontal; whereas the full-range gravity effect over the Z-axis measures is about ±9%, in the effective range for the proposed task, which was approximately ±3%, which is not compensated, and the averages of the effective range values are then used as the zero offsets.

**Table 1:** Offsets and effects of the gravity in non-contact sensor readings (raw values).

Sensor Elevation	X	Y	Z
$0^{\circ }$ (Horizontal)	−232	440	−103
$90^{\circ }$ (Upward)	−256	441	−222
$-90^{\circ }$ (Downward)	−258	439	132
$-180^{\circ }$ (Horizontal)	−282	436	−100
$8^{\circ }$ (Max. Effective Elev.)	−231	440	−120
$-22^{\circ }$ (Min. Effective Elev.)	−233	439	−53
Maximum Gravity Effect	±2.06%	±0.19%	±9.05%
Effective Gravity Effect	±0.065%	±0.015%	±3.16%
Effective Zero Offsets	−232	440	−86

In theory, a force applied in the direction of one load cell should not affect the others. In practice, the cross-talk is a common and undesired effect. To calibrate the outputs and compensate the cross-talk effect, a 750-g load was applied to the sensor end. In the vertical and horizontal position, the effects of the weight along all axes were recorded. Then, with the help of a pulley, the same force was carefully applied to the sensor ball in the horizontal direction. The example measures and performance (obtained by dividing the reading due to an extraneous load by the full scale output for that same channel in %) are shown in Table 2.

**Table 2:** Example of sensor readings for a full-scale load in each direction (cross-talk performance in %).

Output Axis	$F_{x}$	$F_{y}$	$F_{z}$
$O_{x}$	1227	18	63
	(100%)	(1.45%)	(5.95%)
$O_{y}$	64	1300	−45
	(5.22%)	(100%)	(4.25%)
$O_{z}$	41	9	−1058
	(3.34%)	(0.71%)	(100%)

Thus, the sensor sensitivity matrix *K* Equation (7) can be built by dividing the cross-talk performance data by the full-scale load. Evaluating $K^{-1}$ once, the scaling and cross-talk compensation can be computed for each sensor as $\hat{F}=K^{-1}O$, where *O* is the offset-compensated sensor reading.

(7) $$K=\left(\begin{array}{ccc} 1.636 & 0.024 & 0.084\\ 0.085 & 1.733 & -0.060\\ 0.054 & 0.012 & -1.410 \end{array}\right);\;K^{-1}=\left(\begin{array}{ccc} 0.610 & -0.009 & 0.036\\ -0.029 & 0.577 & -0.026\\ 0.023 & 0.004 & -0.707 \end{array}\right)$$

Sources of error include mechanical inaccuracies, electrical noise and the haptic accelerations. However, to minimize the delay in the teleoperated control loop, no low-pass filters have been used.

### 3.4. In Vitro Tissues

As a physical model (phantom) of the patient’s tissues, a set of 15 different artificial organs with different numbers of hard inclusions was built. The phantoms were built with pieces of low density (30 kg/m${}^{3}$) flexible polyurethane foam measuring 18 cm long with a 2×2 cm square section. A latex envelope provided a more realistic look with the desired opacity, colour and texture, but also provides the friction necessary for a stable grasp with two fingers. The hard inclusions are created with 14-mm rigid balls. Five phantoms per the number of inclusions (zero to two) were made (15 phantoms in total). The number of lumps could not be determined by visual inspection.

**Figure 7:**
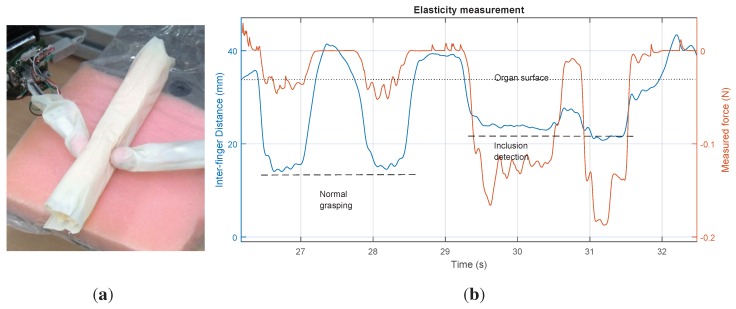
Physical gripper with force sensors performing the intraoperative diagnostic procedure on a physical model of the patient’s organ. (**a**) Physical model of the soft tissue (phantom); (**b**) distance between the centres of the fingertips and measured forces in one finger are shown. The left half shows normal contact. The right half shows the detection of a hard lump.

To identify the phantom impedance during grasping operations with and without lumps, experiments were performed to illustrate different behaviours, as can be seen in Figure 7b.

**Figure 8:**
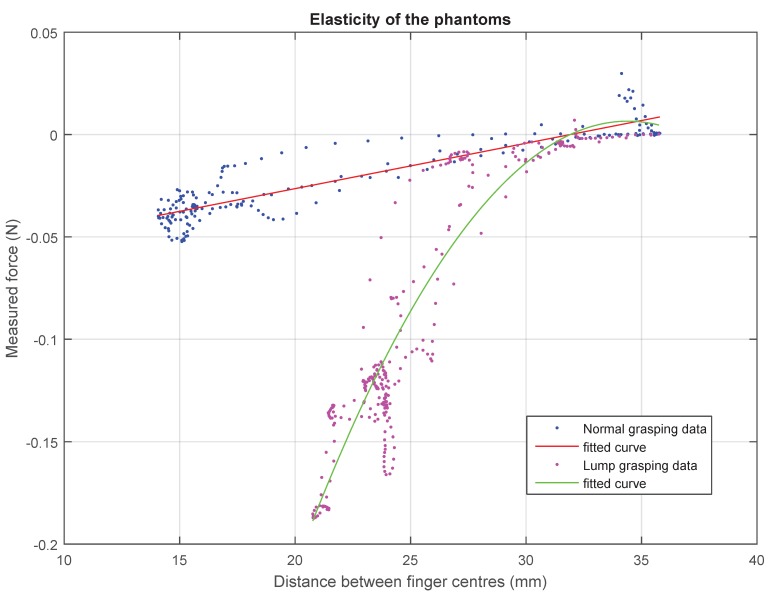
Displacement-force plots of the foam phantoms, showing different behaviours: a linear model describes the homogeneous tissue, and a quadratic equation is used to describe the elasticity of the tissue with hard inclusions.

The elasticity of the phantom in normal conditions can be modelled as a constant value $K_{ph}$ = 2.349 N/m. However, in non-homogeneous sections with hard inclusions, this linear behaviour only applies to the first millimetres and can be better described by a second order polynomial regression, as shown in Figure 8.

### 3.5. Set of Virtual Sensor Configurations

A total of five different sensor configurations for the virtual gripper are proposed and summarized in Table 3. The set of configurations range from six to zero force sensors. The purpose of this set is to evaluate the performance of the teleoperated gripper for a diagnostic task. As using more sensors makes for a more expensive and bulky surgical device, the ideal device has to balance the performance against the number of physical sensors. A sensor-less configuration is possible thanks to the visual feedback. Laparoscopic surgeons have little force feedback, so they are trained to perform surgery relying mostly on video feedback from the camera.

**Table 3:** Set of five virtual sensor configurations for the two-fingered virtual grasper used in the experiments.

Virtual Sensor	Matrix *S*	Description
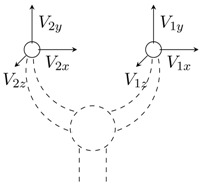	$\left(\begin{array}{cccccc} 1 & 0 & 0 & 0 & 0 & 0\\ 0 & 1 & 0 & 0 & 0 & 0\\ 0 & 0 & 1 & 0 & 0 & 0\\ 0 & 0 & 0 & 1 & 0 & 0\\ 0 & 0 & 0 & 0 & 1 & 0\\ 0 & 0 & 0 & 0 & 0 & 1 \end{array}\right)$	1. Unconstrained. Six-axis (3+3) force sensor. All six forces are simply rotated to the global reference system and fed back to the master haptics. Full rank matrix.
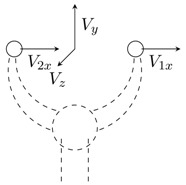	$\left(\begin{array}{cccccc} 1 & 0 & 0 & 0 & 0 & 0\\ 0 & \frac{1}{2} & 0 & 0 & \frac{1}{2} & 0\\ 0 & 0 & \frac{1}{2} & 0 & 0 & \frac{1}{2}\\ 0 & 0 & 0 & 1 & 0 & 0\\ 0 & \frac{1}{2} & 0 & 0 & \frac{1}{2} & 0\\ 0 & 0 & \frac{1}{2} & 0 & 0 & \frac{1}{2} \end{array}\right)$	2. Independent closing force and averaged wrist sensors. Sensors in the *Y* and *Z* direction have been unified (average) to emulate a single sensor located on the grasper body. This configuration saves two physical sensors. Matrix Rank 4.
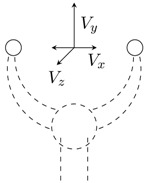	$\left(\begin{array}{cccccc} \frac{1}{2} & 0 & 0 & -\frac{1}{2} & 0 & 0\\ 0 & \frac{1}{2} & 0 & 0 & \frac{1}{2} & 0\\ 0 & 0 & \frac{1}{2} & 0 & 0 & \frac{1}{2}\\ -\frac{1}{2} & 0 & 0 & \frac{1}{2} & 0 & 0\\ 0 & \frac{1}{2} & 0 & 0 & \frac{1}{2} & 0\\ 0 & 0 & \frac{1}{2} & 0 & 0 & \frac{1}{2} \end{array}\right)$	3. Averaged forces. Three virtual force gauges. Sensors in all directions have been unified (average) to emulate a single sensor located on the grasper body. This configuration saves two physical sensors. Matrix Rank 3.
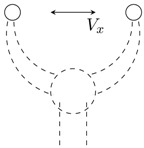	$\left(\begin{array}{cccccc} \frac{1}{2} & 0 & 0 & -\frac{1}{2} & 0 & 0\\ 0 & 0 & 0 & 0 & 0 & 0\\ 0 & 0 & 0 & 0 & 0 & 0\\ -\frac{1}{2} & 0 & 0 & \frac{1}{2} & 0 & 0\\ 0 & 0 & 0 & 0 & 0 & 0\\ 0 & 0 & 0 & 0 & 0 & 0 \end{array}\right)$	4. Grasping force only. A single sensor is used for the measurement of the closing force. Matrix Rank 1.
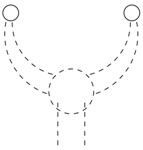	$\left(\begin{array}{cccccc} 0 & 0 & 0 & 0 & 0 & 0\\ 0 & 0 & 0 & 0 & 0 & 0\\ 0 & 0 & 0 & 0 & 0 & 0\\ 0 & 0 & 0 & 0 & 0 & 0\\ 0 & 0 & 0 & 0 & 0 & 0\\ 0 & 0 & 0 & 0 & 0 & 0 \end{array}\right)$	5. No force sensor No force feedback is provided. Visual-only feedback is used. This configuration is provided for performance comparison. Matrix Rank 0.

## 4. Experiments

The goal of the designed experiments was to find out how the different slave robot configurations (with different numbers of sensors) are related to the task performance (completion time and number of errors). The number of errors was obtained as the absolute value of the difference between the actual and detected number of inclusions in an organ.

A two-fingered slave robot end-effector with force feedback is proposed with a set of five different configurations described in Table 3. Each configuration has a different number of virtual sensors. The number of these sensors should be minimized, and the position optimized, without a significant effect on the task performance.

Laboratory trials with 23 volunteers were carried out under ethics approval from the Engineering School of the University of Reading. The experiments were done by 21 non-clinical subjects and two subjects who were regular users of the da Vinci surgical robot (Intuitive Surgical Inc., Mountain View, CA, USA). Each volunteer was provided with an information sheet and an informed consent form. In addition to quantifying errors, each subject completed a questionnaire on the subjective aspects of the surgical instrument prototype.

Each volunteer executed three trials for each one of the five proposed configurations with a randomly-selected organ phantom. Each time, the user was required to give a diagnosis as the number of inclusions identified inside the organ.

According to the user-centred design methodology, the following data were collected: time taken to complete the task, user error rate and subjective user satisfaction. The grasper positions and forces were recorded every 10 ms for further analysis. The experiment took about 35 min per volunteer. The subjective user satisfaction is based on a rating of how intuitive the user finds each prototype and its confidence level.

## 5. Results and Discussion

**Figure 9:**
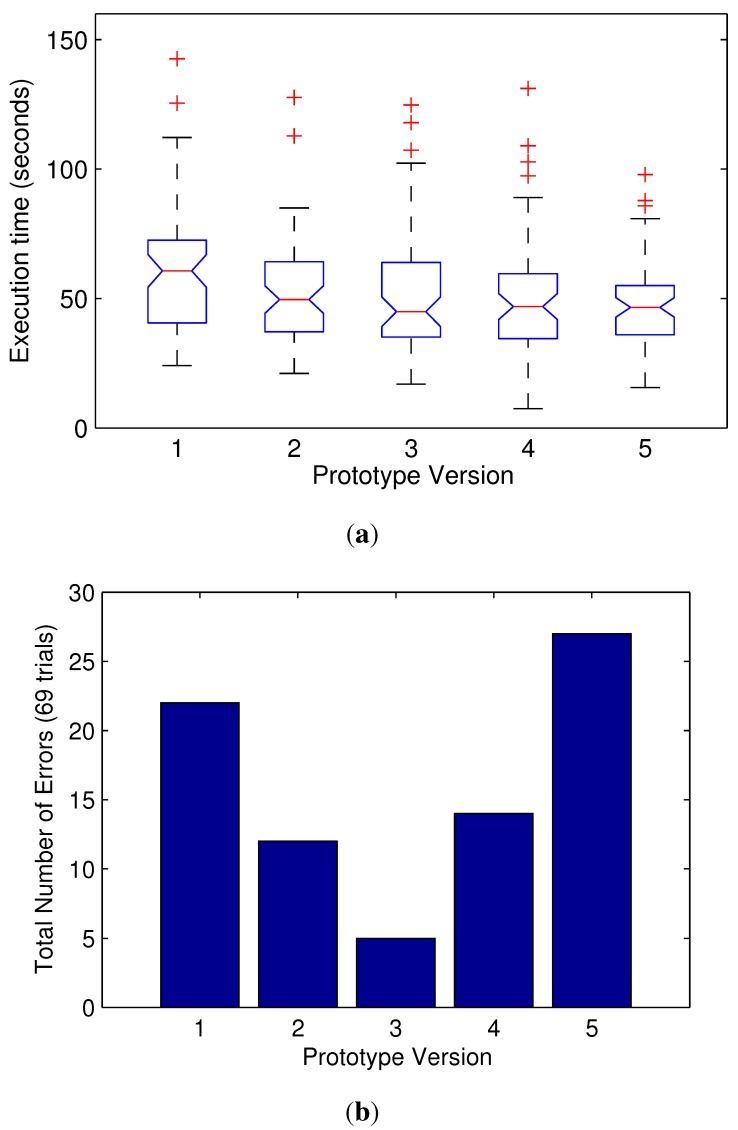
Plots of the different prototypes’ performance in terms of task completion times and number of errors (failure to identify the inclusion count). The central mark is the median, and the edges of the box are the 25th and 75th percentiles. (**a**) Task completion times for the different prototypes; (**b**) total number of errors for each prototype.

The execution times for the performance of the diagnostic task using the prototypes described in Table 3 are represented in Figure 9 in a box plot (the median and a box from the 25th to the 75th percentiles). Outliers are represented by a red cross. They show similar results, but the minimum completion time average is for Prototype Version 3.

The total number of errors gives interesting results: Prototype 3 (with just three sensors) provides much more accurate diagnostics. As expected, the sensor-less Prototype 5 gives a higher number of errors. Unexpectedly, Prototypes 1 and 2 also have a higher number of errors despite having a higher number of sensors (six and five sensors, respectively). This suggests that these additional sensors provide unnecessary information for the proposed task and may distract the user.

**Figure 10:**
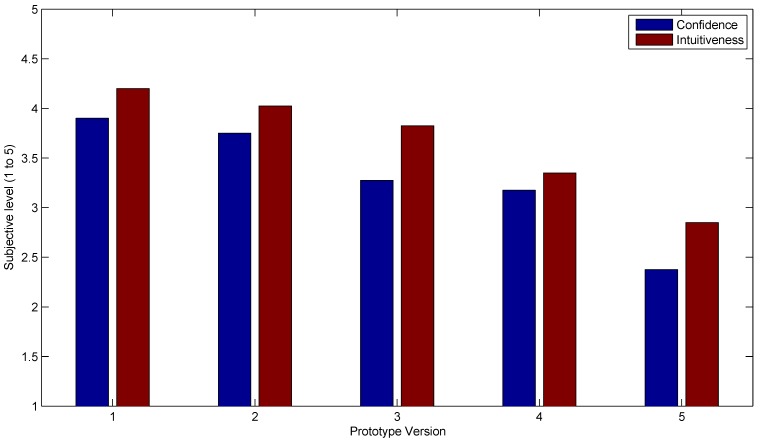
Subjective evaluation of the prototypes in terms of confidence level and intuitiveness, showing a lower rating for a lower sensor count.

The subjective prototype rating is summarized in Figure 10, where the averaged confidence and intuitiveness user ratings for each prototype are shown. Even though the most reliable is Prototype 3, the subjective confidence and intuitiveness values were when more sensors were used.

Although not statistically significant, the results for two of the subjects who were surgeons with direct experience in RAMIS presented shorter times and zero errors in all cases, but the last one (sensor-less prototype) with one error.

As a result, Prototype 3 (with a single closing force sensor in the gripper and two more orthogonal force sensors located in the wrist) seems to be the winning design, because it delivered a lower number of errors with similar execution times, a reduced number of sensors, while keeping a high intuitiveness and confidence rating.

## 6. Conclusions

A prototyping system to evaluate the usability of teleoperated instruments based on the use of virtual sensors has been presented. This system enables more realistic modelling of the final design, because a real physical environment is used to evaluate the prototype.

The proposed method proposes a structure for the modelling of the virtual prototype in terms of the kinematic constraints and virtual sensor matrices. A set of five virtual prototypes of the teleoperated grasper with different sensory configurations has been proposed and modelled.

An experimental test-bed has been designed, used for the evaluation of prototypes of a laparoscopic teleoperated grasper. A general two-fingered master-slave force reflecting system has been implemented and described using widely-available components. The physical general three-axis force sensor on each finger can be used to simulate most of the configurations for the desired prototypes with significant time and cost savings.

The performance of the prototypes with different sensors has been evaluated for an intra-operative diagnostic task in clinical experiments with 23 volunteers performing three lump-detection tasks with each prototype. The number of diagnostic errors, the trajectories and forces were recorded together with the subjective evaluation of each prototype.

The analysis of the objective and subjective data obtained helped to find the optimal design in terms of diagnostic error reduction, the number of physical sensors needed and also user confidence.

This approach has a direct impact on the field of telepresence in surgery, in particular, the development of new force-feedback devices can benefit from this virtual and physical prototyping method.

The virtual prototyping method outlined in this paper enables an assessment of the conflicting pressures of providing high-quality haptic feedback to surgeons and the need to miniaturize the instruments required for endoluminal surgical robotics [24]. The application field of this method is telepresence in surgery, which has been highlighted as an important area of science and technology by the IEEE Technical Committee on Haptics [25].
